# Diagnostic Performance of Intracoronary Optical Coherence Tomography-Modulated Quantitative Flow Ratio for Assessing Coronary Stenosis

**DOI:** 10.1016/j.jscai.2023.101043

**Published:** 2023-05-31

**Authors:** Tianxiao Xu, Wei Yu, Daixin Ding, Chunming Li, Jiayue Huang, Takashi Kubo, William Wijns, Shengxian Tu

**Affiliations:** a Biomedical Instrument Institute, School of Biomedical Engineering, Shanghai Jiao Tong University, Shanghai, China; b The Lambe Institute for Translational Medicine, The Smart Sensors Laboratory and Curam, National University of Ireland, Galway, Ireland; c Hachioji Medical Center, Tokyo Medical University, Tokyo, Japan; d Department of Cardiovascular Medicine, Wakayama Medical University, Wakayama, Japan

**Keywords:** coronary physiology, fractional flow reserve, optical coherence tomography, quantitative flow ratio

## Abstract

**Background:**

A novel method for fast computation of Murray law-based quantitative flow ratio (μQFR) from coregistered angiography and optical coherence tomography (OCT) was recently developed. This study aimed to evaluate the diagnostic performance of this OCT-modulated μQFR (OCT-μFR).

**Methods:**

Patients who underwent coronary angiography, OCT, and fractional flow reserve (FFR) were retrospectively enrolled. μQFR was computed from a single angiographic projection. Subsequently, OCT image pullback was coregistered with the angiogram, and OCT-μFR was calculated based on the coregistered data. The same cut-off value of 0.80 was used for OCT-μFR, μQFR, and FFR to define ischemia.

**Results:**

A paired comparison of OCT-μFR and μQFR was performed in 269 vessels from 218 patients. The mean FFR was 0.81 ± 0.11, and 45.0% of vessels had an FFR ≤0.80. OCT-μFR showed a better correlation with FFR than μQFR (r = 0.83 vs 0.76, *P* = .018) and numerically higher diagnostic performance (area under the curve [AUC] = 0.95 vs 0.92, *P* = .057). Sensitivity, specificity, positive predictive value, negative predictive value, positive likelihood ratio, and negative likelihood ratio for OCT-μFR to identify ischemia-causing stenosis were 89.3%, 93.2%, 91.5%, 91.4%, 13.2, and 0.1, respectively. In addition, OCT-μFR showed significantly higher diagnostic performance compared with μQFR in vessels with suboptimal angiographic image quality (AUC = 0.93 vs 0.87, *P* = .028) and tandem lesions (AUC = 0.94 vs 0.87, *P* = .017).

**Conclusions:**

Computation of OCT-μFR was feasible and accurately identified physiologically significant coronary stenosis with simultaneous morphological assessment. In vessels with suboptimal angiographic image quality or tandem lesions, OCT-μFR had a higher diagnostic performance than angiography-based μQFR.

## Highlights

• OCT-μFR is a novel computational FFR taking advantage of both angiography and OCT.
• OCT-μFR is superior to angiography-based μQFR especially with suboptimal angiographic image quality or tandem lesions.
• OCT-μFR has high usability with incomplete lesion coverage by OCT.

## Central Illustration

**Figure:**
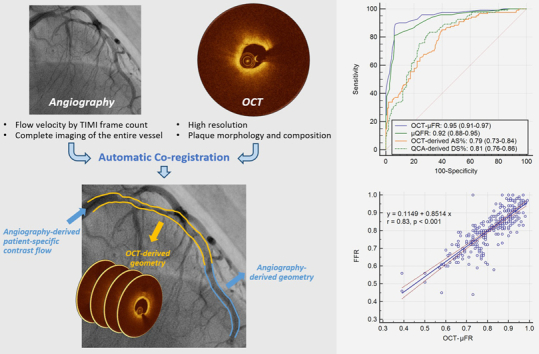

## Introduction

The clinical adoption of physiological assessment of coronary stenosis during the percutaneous coronary intervention (PCI), including fractional flow reserve (FFR), remains heterogeneous and very low worldwide,^1^ despite guideline recommendations.^2^ Recently, computational FFR techniques have been developed as alternatives without using pressure wires or hyperemia-inducing medications.^3^, ^4^, ^5^, ^6^ Quantitative flow ratio (QFR) is the most extensively validated method for fast computation of FFR based on 3-dimensional (3D) angiographic reconstruction and fluid dynamics algorithms.^7^^,^^8^ Recently, the method was upgraded to incorporate Murray bifurcation fractal law in the computation, allowing the computation of QFR from a single angiographic view.^9^ This Murray law-based QFR (μQFR) was shown to have higher usability and similar diagnostic accuracy compared with 2-view-based 3D QFR.^10^^,^^11^ However, in patients with complex coronary anatomy, it might still be challenging to obtain a single angiographic view with minimal overlap and foreshortening along the entire vessel, which might impair the reliability of computational approaches from optical coherence tomography (OCT) as well as from angiography.

As a high-resolution intracoronary imaging technique, OCT allows for detailed characterization of the coronary artery morphology and plaque composition, providing a precise reconstruction of vessel dimensions that is crucial for the computation of FFR.^12^^,^^13^

In this study, we developed a new approach for fast computation of μQFR from coregistered angiography and OCT images, taking advantage of both imaging modalities and providing a simultaneous morphological and physiological assessment of the entire interrogated vessel. Furthermore, we aimed to evaluate the diagnostic performance of this OCT-modulated μQFR (OCT-μFR) compared with μQFR using wire-based FFR as the reference standard.

## Materials and methods

### Study design and patient population

This is a retrospective single-center observational study to evaluate the diagnostic performance of OCT-μFR compared with μQFR in predicting physiologically significant coronary stenosis. Patients undergoing angiography, OCT, and FFR measurements between August 2011 and October 2018 at Wakayama Medical University Hospital (Wakayama, Japan) were enrolled for the current post hoc analysis. This patient population has been reported in a previous study investigating the diagnostic accuracy of OCT-based FFR (optical flow ratio [OFR]) without coregistration with coronary angiogram.^14^ Vessels with balloon predilatation prior to OCT imaging were excluded. μQFR was computed from a single angiographic projection with the best exposure of lesion severity for all enrolled patients except for (1) angiograms with poor contrast filling insufficient for thrombolysis in myocardial infarction (TIMI) frame count; (2) severe overlap at the interrogated lesions; (3) severe tortuous vessels. Subsequently, the OCT image pullback was coregistered with the angiogram using validated algorithms,^15^ and OCT-μFR analysis was performed unless the OCT image quality precluded visualization of the coronary lumen or showed severe image artifact. Finally, paired μQFR and OCT-μFR analysis results were excluded from comparison with wire-based FFR if any of the following was present: (1) myocardial bridge in the interrogated vessel; (2) presence of vessel spasm or injury during OCT imaging; (3) substantial thrombosis identified by OCT; (4) bypass grafting of the interrogated vessel; (5) unacceptable quality of the FFR pressure tracings, including pressure drift, poor quality of the signals and lack of hyperemic response after administration of vasodilators.

The diagnostic performance of OCT-μFR and μQFR were further compared in various subgroups, including vessels with suboptimal angiographic image quality, incomplete lesion coverage by OCT pullback, tandem lesions, bifurcations lesions, and with prior myocardial infarction (MI). Angiographic image quality was deemed as suboptimal if the stenotic segment overlapped with adjacent arteries or when the lumen boundaries at the stenotic segment were blurred because of suboptimal contrast filling. Tandem lesions were defined as 2 or more stenoses that were separated by angiographically normal segments. Bifurcation lesions were defined as stenoses at branch ostia. Prior MI was defined as an MI that occurred more than 1 month before index angiography. Incomplete lesion coverage by OCT was identified if the pullback failed to cover lesions with angiography-based percent diameter stenosis (DS%) ≥20% by visual estimation, of which possible reasons were: (1) Some operators focused on the most severe lesions but ignored mild lesions when performing OCT. (2) The OCT pullbacks in this study were limited to 54 mm and could not cover long lesions in a single pullback. In the present study, vessels with incomplete lesion coverage by OCT could be analyzed using the coregistration of OCT with angiograms, whereas they had been excluded from OFR analysis in the previous study.^14^

### Coronary angiography, FFR, and OCT measurement

Coronary angiography, FFR, and OCT were performed according to local clinical standards. Of note, angiograms were recorded at 15 frames/seconds. FFR pressure was measured with the pressure sensor located at the distal end of the interrogated vessel. OCT pullbacks were imaged at a rotational speed of 100 or 180 frames/sec. Details of imaging were described in Supplemental Appendix S1.

### μQFR and OCT-μFR analysis

All angiographic and OCT images were sent to an independent academic core laboratory (CardHemo, Med-X Research Institute, Shanghai Jiao Tong University, Shanghai, China) for data screening and computational analysis. μQFR and OCT-μFR analysis was performed by a qualified analyst and checked by another experienced analyst, who were both blinded to the FFR values, using the AngioPlus core software version V3 (Pulse Medical).

Firstly, μQFR was computed from a single angiographic projection with optimal exposure of the lesions using the methodology described previously.^9^^,^^16^ In summary, the lumen contours of both the interrogated vessel and major side branches were delineated. Reference vessel diameter was reconstructed considering the nondiseased vessel diameter from healthy segments and the step-down phenomenon across bifurcations based on Murray bifurcation fractal law. TIMI frame count-based contrast-flow velocity was derived automatically and converted into hyperemic flow velocity (HFV). Finally, pressure drop was calculated based on fluid dynamic equations with HFV, the next μQFR pullback along the interrogated vessel was computed, and the μQFR value at the distal end of each side branch was also available.

Subsequently, a selected OCT pullback image was coregistered with the angiogram using an automatic coregistration algorithm,^15^ matching each OCT cross-section on the angiographic centerline. The coregistration accuracy was carefully checked at fiduciary anatomical landmarks (eg, bifurcation and plaque characteristics). A manual correction was allowed in case of inaccurate coregistration results. From OCT, the contours of both the coronary lumen and side branch ostia were delineated and reconstructed in 3D.^17^ The geometric model of the OCT-interrogated segment was then combined with the remaining proximal and distal segments from angiography. Based on this new geometric model of the entire interrogated vessel, a new reference vessel diameter function incorporating the media size and the step-down phenomenon across bifurcations was quantified. Finally, pressure drop was calculated based on fluid dynamics equations^18^ using the above-mentioned HFV. In the end, OCT-μFR was available for both the interrogated vessel and its side branches.

Assuming the FFR pressure wire was pulled back from the distal position, the μQFR and OCT-μFR values at the distal position of the analyzed vessel were used for comparison with wire-based FFR. The entire procedure for coregistration of the OCT with the angiogram was integrated into a single software that enables simultaneous evaluation of the corresponding angiogram and OCT. In general, analysis time can be estimated at approximately 1 minute for μQFR analysis^9^ and another 1 minute for subsequent OCT-μFR analysis.

### Statistical analysis

Continuous variables were reported as mean ± SD if normally distributed or median [quartiles] if nonnormally distributed. Categorical variables were presented as n (%). The correlation was assessed by the Pearson correlation coefficient. Bland-Altman plot and intraclass correlation coefficient for the absolute values (ICCa) were used to assess the agreement between OCT-μFR and FFR and between μQFR and FFR. A comparison of ICCa was performed using an online tool (https://www.psychometrica.de/correlation.html). F-test was used for comparison of limits of agreement in Bland-Altman plot analysis. The same cut-off value of 0.80 was used for OCT-μFR, μQFR, and FFR to define flow-limiting coronary stenosis,^14^^,^^16^ whereas the cut-off value for DS% and OCT-derived percentage area stenosis was set as 50%^14^^,^^16^ and 70%.^19^^,^^20^ χ^2^ test was used for comparison of relative diagnostic accuracies. Diagnostic performance was evaluated by the area under the curve (AUC) of receiver operating characteristics. The Delong method was used for AUC comparison. All data were analyzed at the per-vessel level. To correct for clustering effects caused by the inclusion of multiple vessels from the same patients, a mixed-effects linear model was applied. Per-patient level analyses were performed. In patients with multiple interrogated vessels, the vessel with the lowest FFR value was considered.

A *P* < .05 was considered statistically significant without prespecified adjustment for multiple testing, and thus statistical results were exploratory. Statistical analysis was performed using MedCalc version 14.12 (MedCalc Software) and Jamovi version 2.2 (The jamovi project).

## Results

### Baseline clinical and lesion characteristics

**Figure 1:**
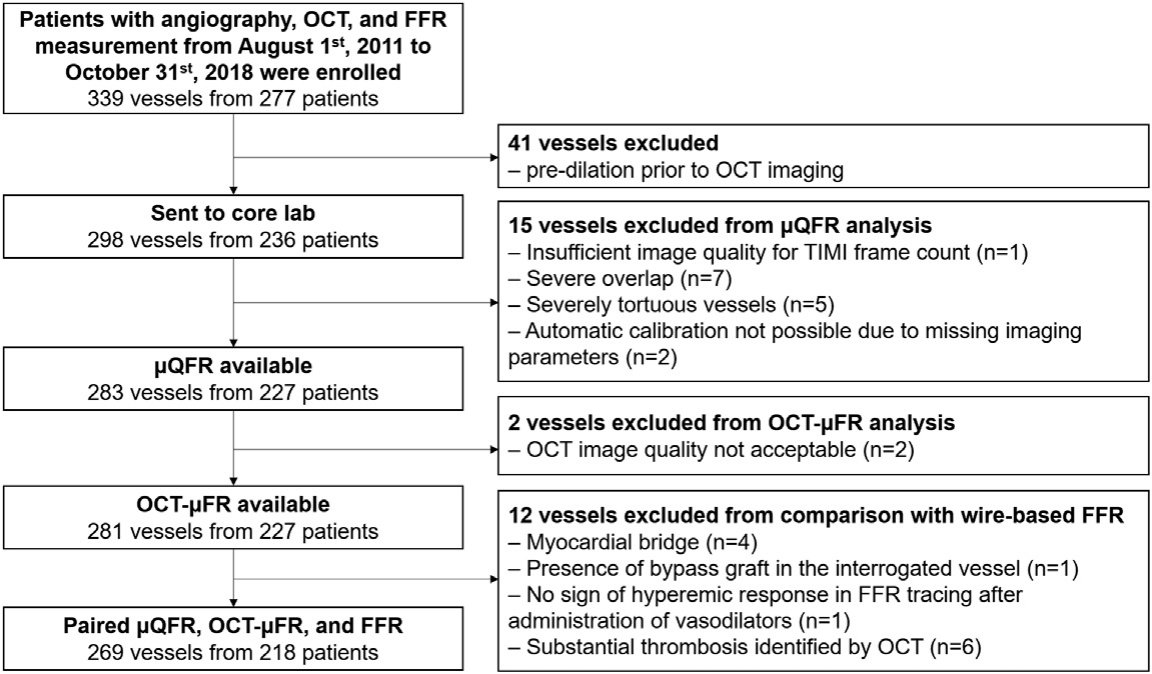
**Study flowchart**. FFR, fractional flow reserve; OCT, optical coherence tomography; OCT-μFR, optical coherence tomography-modulated Murray law-based quantitative flow ratio; μQFR, Murray law-based quantitative flow ratio; TIMI: thrombolysis in myocardial infarction.

A total of 339 vessels from 277 patients were enrolled in the study (Figure 1). Before core laboratory analysis, 41 vessels were excluded because of predilatation prior to OCT imaging. In the core laboratory, 15 vessels were excluded from μQFR analysis, and 2 vessels were further excluded from OCT-μFR analysis based on predefined exclusion criteria. For comparison with wire-based FFR, 12 vessels were excluded. As a result, 269 vessels (79.4%) from 218 patients were available for paired μQFR and OCT-μFR comparison.

**Figure 2:**
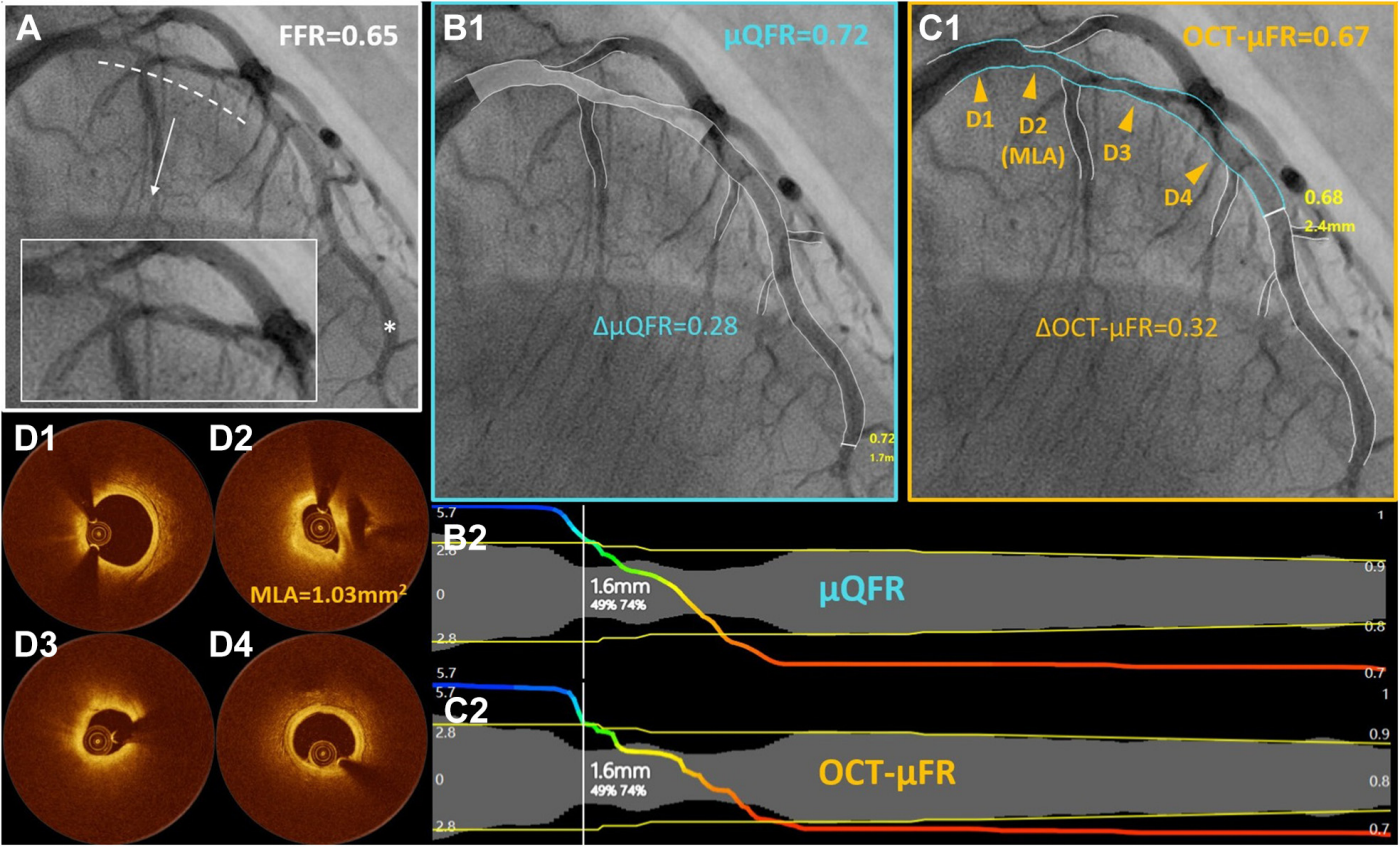
**Representative example of μQFR and OCT-μFR computations.** (**A**) Coronary angiography showed a diffuse lesion in the proximal left anterior descending artery, with vessel overlap and contour blurring in the stenotic segment (dotted line). FFR measured at the asterisk position was 0.65. (**B1**) μQFR analysis based on a single angiographic projection. μQFR value at the asterisk position was 0.72, with a pressure drop along the stenotic segment (ΔμQFR) of 0.28. (**B2**) The μQFR pullback curve along the vessel. (**C1**) OCT-μFR analysis based on the same angiogram and OCT pullback. The OCT image was coregistered with the angiogram, with the segment covered by OCT delineated by the blue line. (**C2**) The OCT-μFR pullback curve along the vessel. Panels (**D1-D4**) corresponded to the 4 positions (orange triangles) in panel C1. Compared to μQFR, the OCT-μFR value at the asterisk position decreased to 0.67, with an increased pressure drop along the stenotic segment (ΔOCT-μFR) of 0.32. FFR, fractional flow reserve; MLA, minimal lumen area; OCT, optical coherence tomography; OCT-μFR, optical coherence tomography-modulated Murray law-based quantitative flow ratio; μQFR, Murray law-based quantitative flow ratio.

Baseline demographic and vessel characteristics are presented in Table 1 and Table 2. The mean and median FFR values of all 269 interrogated vessels were 0.81 ± 0.11 and 0.82 (0.73-0.88), respectively. FFR ≤ 0.80 was identified in 121 (45.0%) vessels. OCT pullbacks from 54 (20.1%) vessels failed to cover all lesions of the interrogated vessels. The angiographic image quality was suboptimal but acceptable in 109 (40.5%) vessels, including moderate overlap and blurring of lumen contours. Representative cases of OCT-μFR and μQFR computations with suboptimal angiographic image quality or incomplete lesion coverage by OCT are shown in Figure 2 and Supplemental Figure S1, respectively.

**Table 1:** Baseline demographic characteristics.

	Patients (N = 218)
Patients with FFR measurement in >1 vessel	43 (19.7%)
Age, y	69 [61-76]
Women	51 (23.4%)
BMI, kg/m^2^	24.1 [22.0-26.1]
Diabetes mellitus	98 (45.0%)
Hypertension	178 (81.7%)
Hyperlipidaemia	158 (72.5%)
Current smoker	44 (20.2%)
Family history of CAD	45 (20.6%)
Previous PCI	145 (66.5%)
Previous CABG	3 (1.4%)
Previous MI	100 (45.9%)
Clinical presentation	
Silent ischemia	109 (50.0%)
Stable angina	56 (25.7%)
Unstable angina	31 (14.2%)
NSTEMI	6 (2.8%)
Others	16 (7.3%)

Data are presented as n (%) or median [quartiles].

BMI, body mass index; CABG, coronary artery bypass graft surgery; CAD, coronary artery disease; FFR, fractional flow reserve; MI, myocardial infarction; NSTEMI, non-ST-segment elevation myocardial infarction; PCI, percutaneous coronary intervention.

**Table 2:** Baseline vessel characteristics.

	Vessels (n = 269)
Interrogated vessel	
Left anterior descending	160 (59.5%)
Diagonal	1 (0.4%)
Left circumflex	43 (16.0%)
Obtuse marginal	1 (0.4%)
Right coronary artery	64 (23.8%)
Minimum lumen area, mm^2^	1.85 [1.24-2.80]
Percent area stenosis, %	65.1 ± 13.8
Percent diameter stenosis, %	42.2 ± 11.6
Subgroups	
Suboptimal angiographic image quality	109 (40.5%)
Incomplete lesion coverage by OCT	54 (20.1%)
Bifurcation lesions	133 (49.4%)
Tandem lesions	58 (21.6%)
FFR	
Mean ± SD	0.81 ± 0.11
Median [quartiles]	0.82 [0.73-0.88]
FFR ≤ 0.80	121 (45.0%)
0.75 ≤ FFR ≤ 0.85	99 (36.8%)

Data are presented as mean ± SD, number (percentage), or median [quartiles]. Minimum lumen area and percent area stenosis were assessed by OCT, whereas percent diameter stenosis was assessed by angiography.

FFR, fractional flow reserve; OCT, optical coherence tomography; SD, standard deviation.

### Correlation and agreement

**Figure 3:**
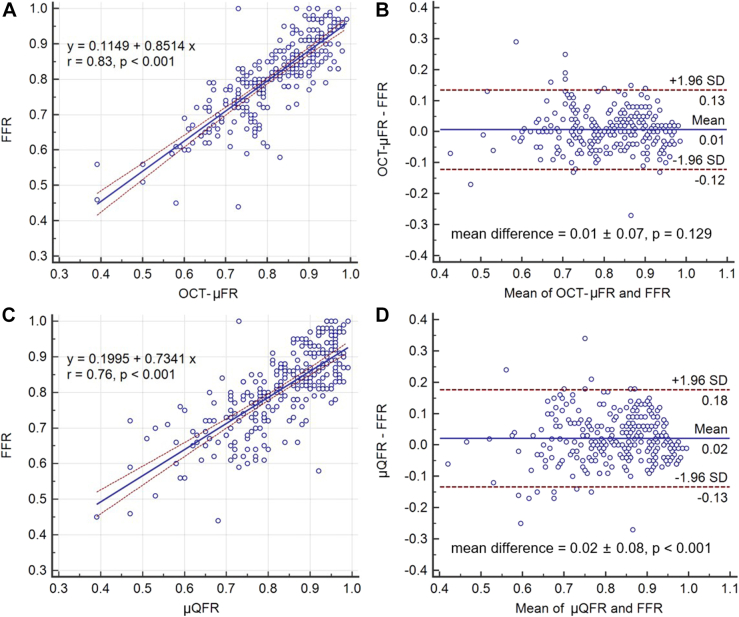
**Correlation and agreement between computational and wire-based FFR. (A)** Correlation between OCT-μFR and FFR. (**B**) Agreement between OCT-μFR and FFR. (**C**) Correlation between μQFR and FFR. (**D**) Agreement between μQFR and FFR. FFR, fractional flow reserve; OCT, optical coherence tomography; OCT-μFR, optical coherence tomography-modulated Murray law-based quantitative flow ratio; μQFR, Murray law-based quantitative flow ratio.

There was significantly better correlation and agreement between OCT-μFR and FFR compared with μQFR and FFR (r = 0.83 vs 0.76, *P* = .018; ICCa = 0.82 vs 0.76, *P* = .018; Figure 3). In addition, OCT-μFR showed better limits of agreement with FFR than μQFR did (standard deviation of the difference = 0.065 vs 0.079, *P* = .002; Figure 3). The results were consistent after correction for within-patient clustering (Supplemental Appendix S2, Supplemental Table S1, and Supplemental Figure S2).

### Diagnostic performance of OCT-μFR, μQFR, OCT, and quantitative coronary angiography

**Figure 4:**
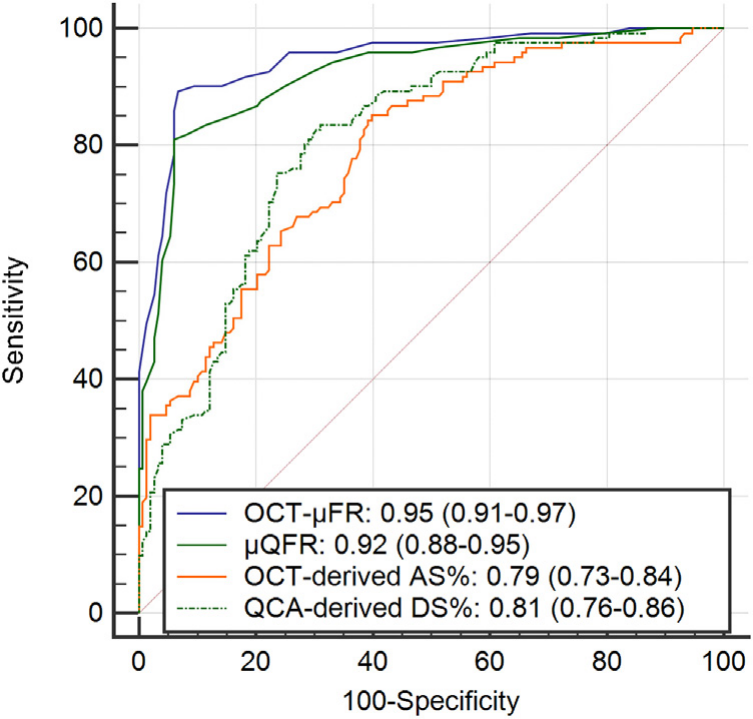
**ROC curves for OCT-μFR, μQFR, OCT-derived AS%, and QCA-derived DS% to identify FFR ≤0.80.** OCT-μFR showed good diagnostic performance in identifying flow-limiting coronary stenosis defined by FFR ≤0.80. AS%, percent area stenosis; DS%, percent diameter stenosis; FFR, fractional flow reserve; OCT, optical coherence tomography; OCT-μFR, optical coherence tomography-modulated Murray law-based quantitative flow ratio; QCA, quantitative coronary angiography; ROC, Receiver operating characteristic; μQFR, Murray law-based quantitative flow ratio.

Using a cut-off of ≤0.80 for identifying physiologically significant stenosis, the AUC for OCT-μFR to identify FFR ≤0.80 did not show statistically significant differences compared with μQFR. However, there was a trend toward marginally higher AUC for OCT-μFR (0.95 vs 0.92, *P* = .057; Figure 4), both being excellent. The overall diagnostic accuracies of OCT-μFR and μQFR were comparable (91.4% vs 88.1%, *P* = .200). Other diagnostic metrics are listed in Table 3. True positives, true negatives, false positives, and false negatives for OCT-μFR to identify FFR ≤0.80 were 108, 138, 10, and 13, respectively. OCT-μFR had a significantly higher AUC than OCT-derived percentage area stenosis (difference = 0.14, *P* < .001) and quantitative coronary angiography-derived DS% (difference = 0.14, *P* < .001). The results were consistent after correction for within-patient clustering (Supplemental Figure S3).

**Table 3:** Diagnostic performance of OCT-μFR, μQFR, OCT-derived AS%, and QCA-derived DS% to identify fractional flow reserve ≤0.80.

	OCT-μFR	μQFR	OCT-derived AS% >70%	QCA-derived DS% ＞50%
Accuracy, % (95% CI)	91.4 (88.1-94.8)	88.1 (84.2-92.0)	71.4 (66.0-76.8)	67.3 (61.7-72.9)
Sensitivity, % (95% CI)	89.3 (82.3-94.2)	81.0 (72.9-87.6)	85.1 (77.5-90.9)	44.2 (35.2-53.5)
Specificity, % (95% CI)	93.2 (87.9-96.7)	93.9 (88.8-97.2)	60.1 (51.8-68.1)	86.2 (79.5-91.2)
PPV, % (95% CI)	91.5 (85.0-95.9)	91.6 (84.6-96.1)	63.6 (55.7-71.0)	72.3 (60.6-82.1)
NPV, % (95% CI)	91.4 (85.7-95.3)	85.8 (79.5-90.8)	83.2 (74.7-89.7)	65.4 (58.3-72.0)
+LR (95% CI)	13.2 (7.2-24.1)	13.3 (7.0-25.2)	2.1 (1.7-2.6)	3.2 (2.1-5.0)
-LR (95% CI)	0.1 (0.1-0.2)	0.2 (0.1-0.3)	0.3 (0.2-0.4)	0.6 (0.5-0.8)
AUC (95% CI)	0.95 (0.91-0.97)	0.92 (0.88-0.95)	0.79 (0.73-0.84)	0.81 (0.76-0.86)

Results are shown as percentages (95% CI) except for AUC and likelihood ratios.

AS%, percent area stenosis; AUC, area under the curves; DS%, percent diameter stenosis; NPV, negative predictive value; OCT, optical coherence tomography; OCT-μFR, optical coherence tomography-modulated Murray law-based quantitative flow ratio; PPV, positive predictive value; QCA, quantitative coronary angiography; μQFR, Murray law-based quantitative flow ratio; +LR, positive likelihood ratio; –LR, negative likelihood ratio.

### Impact of angiographic image quality

The presence of suboptimal angiographic image quality reduced the diagnostic performance of μQFR in predicting FFR ≤ 0.80 (AUC = 0.94 vs 0.87, *P* = .090) but did not influence that of OCT-μFR (AUC = 0.94 vs 0.93, *P* = .789). The diagnostic performance of OCT-μFR was superior to μQFR in the presence of suboptimal angiographic image quality (AUC = 0.93 vs 0.87, *P* = .028) but comparable in vessels with optimal angiographic image quality (AUC = 0.94 vs 0.94, *P* = .879).

### Impact of tandem lesions

A total of 58 (21.6%) vessels had tandem lesions, with a mean FFR of 0.78 ± 0.11. The diagnostic performance of OCT-μFR was superior to μQFR in vessels with tandem lesions (AUC = 0.94 vs 0.87, *P* = .017) but was comparable in vessels without tandem lesions (AUC = 0.94 vs 0.94, *P* = .977).

### Impact of incomplete lesion coverage by OCT

The OCT image pullbacks of 54 (20.1%) interrogated vessels failed to cover all lesions, with a mean FFR of 0.74 ± 0.14. As expected, OCT-μFR had numerically lower AUC (0.95 vs 0.91, *P* = .386) and diagnostic accuracy (93% vs 87%, *P* = .196) in subgroups with complete versus incomplete lesion coverage by OCT, without reaching statistically significant differences.

### Impact of bifurcation lesions and prior MI

The diagnostic performance of OCT-μFR was not significantly different in bifurcation or nonbifurcation lesions (AUC = 0.93 vs 0.95, *P* = .479) and in patients with or without prior MI (AUC = 0.94 vs 0.95, *P* = .944). OCT-μFR and μQFR had comparable diagnostic performance with bifurcation lesions (AUC = 0.93 vs 0.91, *P* = .441) and in patients with prior MI (AUC = 0.94 vs 0.93, *P* = .313).

## Discussion

We developed a new approach for fast computation of FFR from coregistered angiography and OCT imaging, called OCT-μFR, and tested the feasibility and diagnostic performance in comparison with angiography-based μQFR, using wire-based FFR as reference. OCT-μFR enables simultaneous morphological and physiological coronary assessment with coregistration of the 2 image modalities. The main findings of this study (Central Illustration) are summarized as follows: (1) OCT-μFR shows good correlation and agreement with wire-based FFR in patients with a high a priori likelihood of PCI. The correlation and agreement with FFR are significantly better for OCT-μFR than μQFR; (2) Using FFR ≤ 0.80 to define physiologically significant coronary stenosis, OCT-μFR showed a trend toward higher overall diagnostic performance over μQFR; (3) For vessels with suboptimal angiographic image quality or tandem lesions, OCT-μFR is superior to μQFR in identifying physiologically significant stenosis; (4) The diagnostic performance of OCT-μFR remains comparable in vessels with patients with prior MI or bifurcation lesions; (5) OCT-μFR has high usability in vessels with incomplete lesion coverage by OCT.

**Central Illustration:**
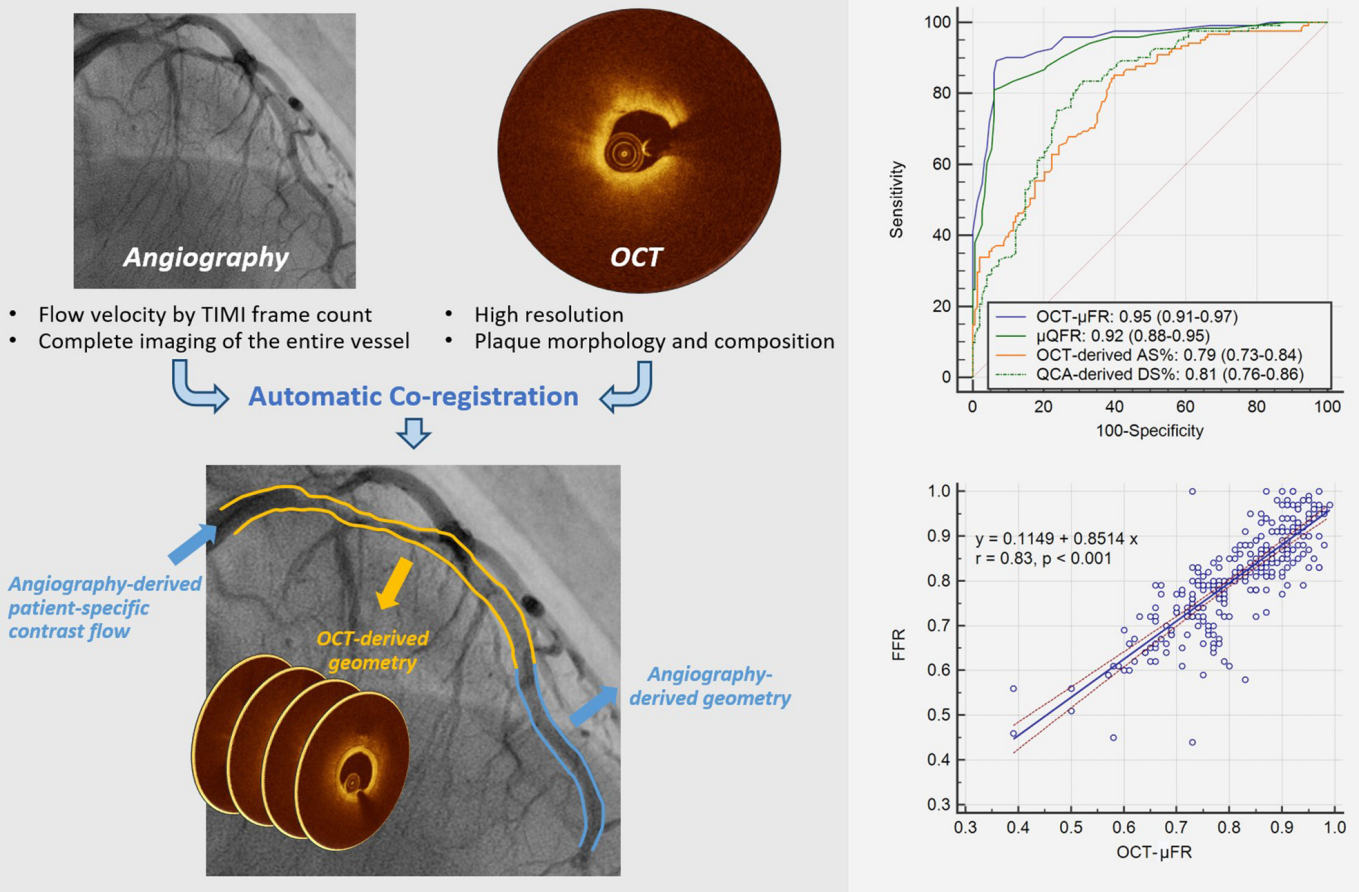
OCT-μFR derived from angiography and OCT for assessing coronary stenosis. OCT-μFR is a fast computational FFR method with improved accuracy in lumen geometry reconstruction and downstream flow estimation from angiography and OCT, esulting in high diagnostic performance (AUC = 0.95) and correlation with FFR (r = 0.83). Moreover, it also provides a simultaneous assessment of coronary morphology and physiology. AS%, percent area stenosis; AUC, area under the curves; DS%, percent diameter stenosis; FFR, fractional flow reserve; OCT, optical coherence tomography; OCT-μFR: OCT-modulated Murray law-based quantitative flow ratio; QCA, quantitative coronary angiography; μQFR: Murray law-based quantitative flow ratio; TIMI: thrombolysis in myocardial infarction.

The superior diagnostic performance of OCT-μFR in predicting wire-based FFR relies on improved accuracy in lumen geometry reconstruction and downstream flow estimation. OCT provides detailed plaque information for selecting nondiseased segments for reference lumen diameter reconstruction. Instead of a fixed-flow model as used in the OFR algorithm, patient-specific contrast flow from angiography is used to estimate flow velocity and is combined with reference lumen geometry for estimation of downstream myocardial flow.

Computational FFR methods from coronary angiography have been extensively validated with good diagnostic performance^5^^,^^21^^,^^22^ and prognostic value.^23^ The recent FAVOR III China trial further demonstrated that the QFR-guided strategy of lesion selection improved 1-year and 2-year clinical outcomes compared with standard angiography guidance.^24^^,^^25^ Nevertheless, the accuracy of angiography-based FFR computational methods depends to some extent on the angiographic image quality.^21^ The common intrinsic limitations of angiography (ie, overlap, foreshortening, poor vessel filling, and low frame rate) restrict the reconstruction precision. It is not always straightforward to acquire an angiographic projection without them, especially in patients with complex coronary anatomy. If the stenotic segments are overlapped with adjacent arteries or are blurred because of suboptimal contrast filling, the lumen boundary is more ambiguous, resulting in lower reconstruction precision and reduced μQFR accuracy. Instead, OCT imaging provides high-resolution and precise lumen geometry, with anticipated improved accuracy of OCT-μFR computation. This is further supported by our finding that OCT-μFR had significantly better diagnostic performance compared with μQFR in the presence of suboptimal angiographic image quality. Additionally, in vessels with tandem lesions, vessel overlap or foreshortening is more likely to result in less accurate coronary artery reconstruction from the single angiographic view. By contrast, OCT provides detailed plaque information, contributing to a more accurate evaluation of coronary lumen and reference vessel diameter.

Several OCT-derived FFR methods have been reported previously. Ha et al^26^ proposed FFR_OCT_ based on computational fluid dynamics and showed good diagnostic performance in predicting FFR ≤ 0.80 (AUC = 0.93). The overall analysis lasted for 10 minutes, and different software applications were needed for 3D reconstruction, meshing, and computational fluid dynamics simulation. Seike et al^27^ developed OCT-FFR based on fluid dynamics equations, with a high correlation with FFR (r = 0.89) and shortened computational time. Cha et al^28^ developed a random-forest-based machine-learning FFR (ML-FFR) and showed good correlation and diagnostic concordance with FFR (r = 0.79, AUC = 0.91). The above methods used a main branch vessel model without accounting for side branches. In general, further clinical validation of these alternative approaches is desirable. We previously developed OFR based on fluid dynamic equations incorporating flow division in bifurcations. OFR has been validated with good performance in de novo lesions, in-stent restenosis, and vessels immediately after PCI,^14^^,^^29^, ^30^, ^31^ with an overall diagnostic accuracy of 91% in a recent individual patient-data meta-analysis pooling 626 vessels from 574 patients.^32^

Compared with OFR, OCT-μFR has greater usability and provides a more comprehensive physiological analysis of the entire interrogated vessel. Cases with incomplete lesion coverage by OCT, if present, were not included in the OFR assessment; thus, the usability of OFR was mainly impaired, especially in retrospective studies without a dedicated image acquisition protocol. By means of OCT-μFR, the usability of the functional evaluation was improved. In this study, 54 more cases with incomplete lesion coverage by OCT were successfully analyzed, which were excluded from OFR analysis in the previous study using the same population.^14^ Encouragingly, the overall diagnostic performance of OCT-μFR was independent of the presence of incomplete lesion coverage by OCT (*P* = .386). Additionally, instead of using generic fixed-flow velocity in the OFR algorithm because of the lack of flow information in OCT, patient-specific contrast flow can be calculated from angiography and used as a boundary condition in OCT-μFR computation. This is important for accurate pressure drop estimation, as the FAVOR pilot study demonstrated that QFR based on the contrast-flow model showed significantly higher diagnostic accuracy than based on the fixed-flow model.^7^

OCT-μFR also provides a simultaneous intravascular assessment of coronary plaque and stent morphology coregistered with angiography. It is valuable to combine computational FFR with plaque morphology, which can significantly improve the predictive value of cardiovascular events.^33^ As OCT is superior to angiography in evaluating intrastent abnormalities (stent malapposition, underexpansion, etc) in post-PCI settings,^34^^,^^35^ OFR was shown capable of informing an overall detailed assessment of stenting results^31^ and had high in-stent correlation with FFR assessments.^30^ These features are also available from OCT-μFR analysis and will be addressed in future studies. Of note, the coregistration of angiography and OCT for OCT-μFR computation was achieved without the need for simultaneous filming of the optical sensor during the acquisition of the angiography.^15^ This is favorable because the routine diagnostic workflow in the catheterization laboratory remains unchanged.

### Limitations

This study has several limitations. Because of the retrospective nature of this study, the location of the pressure sensor during FFR measurement was not recorded in all patients, and the values at the most distal end of the analyzed vessels were used for OCT-μFR and μQFR. Thus, we cannot completely rule out that FFR and OCT-μFR were compared at different locations in some instances, which may downgrade their concordance. A prospective study with recorded wire locations should be performed in order to quantify the incidence and impact of mismatch in the distal measurement boundary. Of note, in a previously reported comparative study, OCT-μFR showed indeed numerically comparable diagnostic performance with OFR, but study populations differ from the current study.^14^ The present study enrolled 54 more challenging vessels. As a result of this, the current study demonstrates increased usability of OCT-μFR compared to OFR. Nevertheless, future prospective head-to-head comparisons between OCT-μFR and OFR are desirable.

For the cases with 2 or more OCT pullbacks, the pullback showing the lesion with the highest DS% was selected for OCT-μFR analysis because the current software only supports the fusion of 1 OCT pullback with 1 angiographic image. In the current workflow, μQFR analysis is the prerequisite of OCT-μFR analysis, so OCT-μFR and μQFR analyses were not blinded to each other. All computational physiology analyses were performed offline in the core laboratory. Future studies investigating the in-procedure utility of OCT-μFR are warranted.

## Conclusion

The fast computation of FFR from coregistered angiography and OCT, named OCT-μFR, was feasible and accurately identified physiologically significant coronary stenosis with simultaneous morphological assessment. In addition, in vessels with suboptimal angiographic image quality or tandem lesions, OCT-μFR had a higher diagnostic performance than angiography-based μQFR.
