# Supplementary material for: Diagnostic Performance of Intracoronary Optical Coherence Tomography-Modulated Quantitative Flow Ratio for Assessing Coronary Stenosis
Source: J Soc Cardiovasc Angiogr Interv. 2023 May 31;2(5):101043. doi: 10.1016/j.jscai.2023.101043 (PMC11308763; doi:10.1016/j.jscai.2023.101043)
Supplement: Supplemental Appendix [file mmc1.docx]

# Supplementary Appendix 1: Coronary angiography, FFR, and OCT

Coronary angiography was performed with a 5 or 6 Fr guiding catheter by a transfemoral or transradial approach. Coronary angiograms were acquired at 15 frames/sec (Allura Xper FD 10; Philips Healthcare, Best, the Netherlands). Contrast media (Omnipaque 350 Injection; Daiichi Sankyo Co. Ltd., Tokyo, Japan) was injected at a rate of 2-4 mL/sec for approximately 2-3 sec.

FFR was performed in vessels with 30% to 90% diameter stenosis by visual estimation on the angiograms. FFR pullbacks were manually performed with a 0.014-inch pressure wire (St. Jude Medical, Uppsala, Sweden) or PrimeWire Prestige (Philips Volcano, San Diego, CA, USA), during steady-state maximal hyperaemia. A continuous intravenous infusion of 150 μg/kg/min adenosine 5'-triphosphate was given to induce maximal hyperaemia. The examination with a pressure drift exceeding 3 mmHg at the end of the pullback was unacceptable and repeated. Analysis of all FFR pressure tracings was performed at the Wakayama Medical University Hospital, using the minimal and stable position during hyperaemia for FFR reading.

OCT pullbacks were obtained at a frame rate of 100 or 180 frames/sec using frequency-domain OCT systems (ILUMIEN™ or OPTIS™; Abbott, St. Paul, MN, USA), with the Dragonfly or Dragonfly DUO catheter.

**Supplementary Appendix 2:** **Patient-level analysis considering clustering effect**

To examine and correct for the clustering effect caused by inclusion of multiple vessels from the same patients, the mixed-effects linear model was applied, introducing patient identification as a random effect, and the model was fitted with random intercepts. The statistical results (**Supplementary Table 1**) were consistent with the linear regression result. Furthermore, the low Intraclass correlation (ICC) indicated that there was a low clustering effect, which means that within-patient homogeneity had limited influence on the correlation of OCT-μFR or μQFR with FFR.

To further explore the potential effect of including multiple vessels in the same patients, per-patient level analyses were performed. In patients with multiple interrogated vessels, the vessel with the lowest FFR value was considered. At patient-level, the correlation and limits of agreement with FFR were significantly better for OCT-μFR than for μQFR (r = 0.80 versus 0.73, p = 0.039; SD of the difference = 0.068 versus 0.084, p = 0.003; **Supplementary Figure 2**). Overall AUC for OCT-μFR to identify FFR≤0.80 was significantly higher than for μQFR (0.94 versus 0.91, p = 0.021; **Supplementary Figure 3**). These patient-level results were consistent with vessel-level analysis.

**Supplementary Table 1: Mixed-effect model linear analysis**

|  | β (95% CI) | Intercept (95% CI) | Random Component ICC |
| --- | --- | --- | --- |
| OCT-μFR | 0.855 (0.785-0.925) | 0.112 (0.055-0.169) | 0.073 |
| μQFR | 0.736 (0.660-0.812) | 0.198 (0.134-0.261) | 0.062 |

β and intercept are the estimated coefficients of the mixed-effect linear model. Random component ICC indicates how different the observations from the same cluster and different clusters are. ICC: intraclass correlation coefficient; OCT: optical coherence tomography; μQFR: Murray law-based quantitative flow ratio; OCT-μFR: OCT-modulated Murray law-based quantitative flow ratio.

**Supplementary Figure 1:** **Representative example of μQFR and OCT-μFR computations with incomplete lesion coverage by OCT.**

**
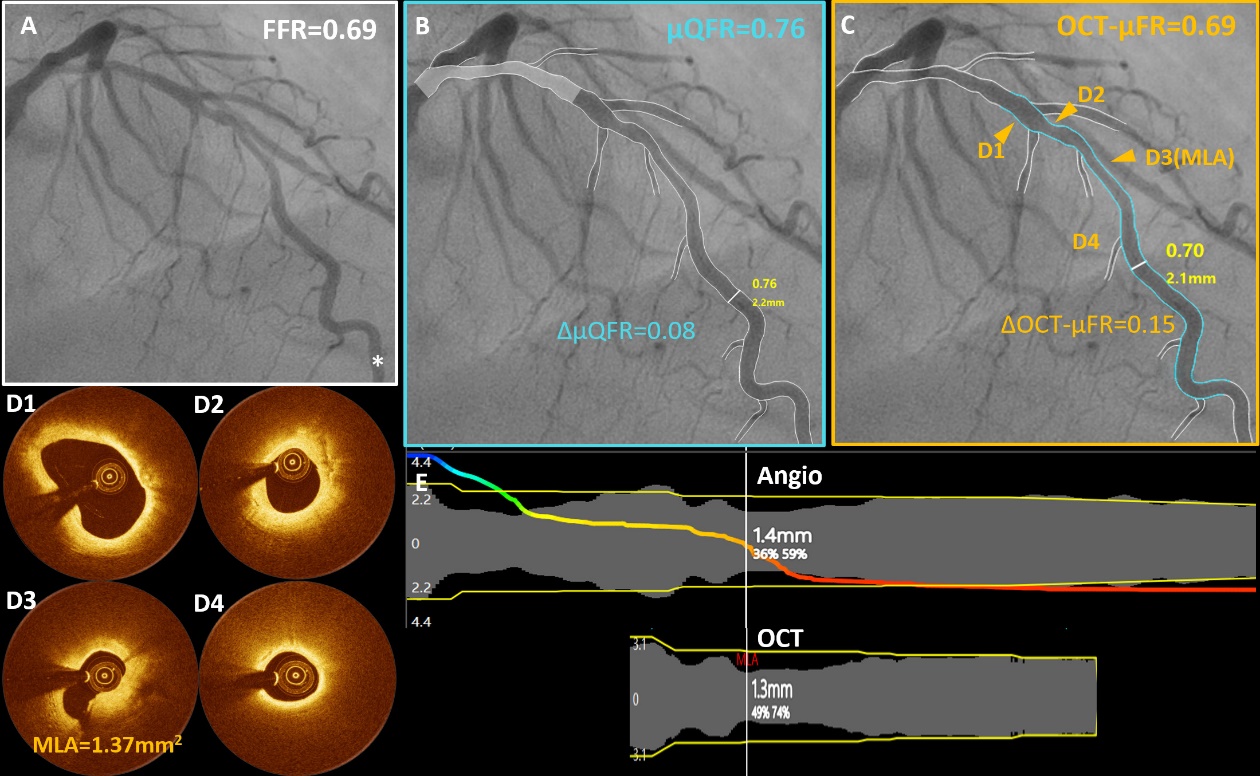
**

(A) Coronary angiography showed a stenotic proximal left anterior descending artery. FFR measured at the asterisk position was 0.69. (B) μQFR analysis based on a single angiographic projection. μQFR value at the asterisk position was 0.76, with a pressure drop along the OCT-covered segment (ΔμQFR) of 0.08. (C) OCT-μFR analysis based on the same angiogram and OCT pullback. The OCT image was co-registered with the angiogram, with the OCT-covered segment delineated by the blue line. Panels D1 to D4 corresponded to the 4 positions (orange triangles) in panel C. (E) The OCT-μFR pullback curve along the vessel and the corresponding OCT pullback. Compared to μQFR, the OCT-μFR value at the asterisk position decreased to 0.69, with increased pressure drop along the OCT-covered segment (ΔOCT-μFR) of 0.15. The pressure drop (ΔOCT-μFR) along the proximal OCT-uncovered segment was 0.16.

FFR: fractional flow reserve; LAD: left anterior descending artery; MLA: minimal lumen area; OCT: optical coherence tomography; μQFR: Murray law-based quantitative flow ratio; OCT-μFR: OCT-modulated Murray law-based quantitative flow ratio.

**Supplementary Figure 2: Patient-level correlation and agreement between computational and wire-based FFR**


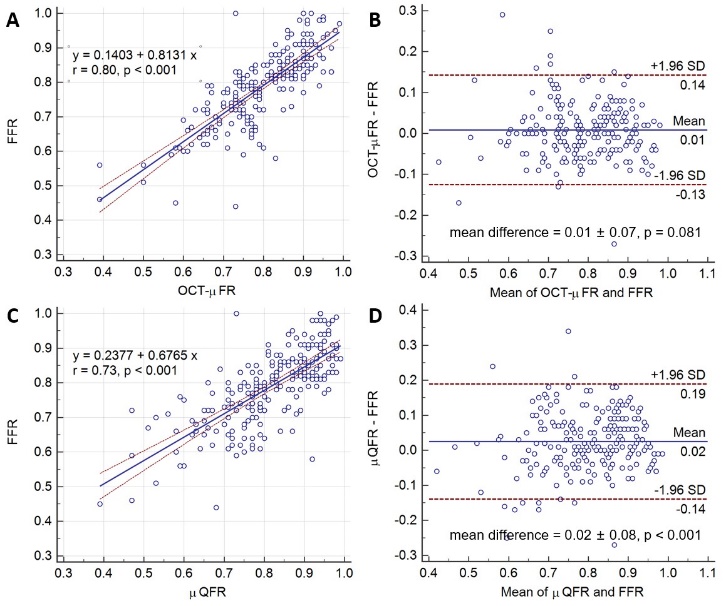


A) Correlation between OCT-μFR and FFR. B) Agreement between OCT-μFR and FFR. C) Correlation between μQFR and FFR. D) Agreement between μQFR and FFR.

FFR: fractional flow reserve; OCT: optical coherence tomography; μQFR: Murray law-based quantitative flow ratio; OCT-μFR: OCT-modulated Murray law-based quantitative flow ratio.

**Supplementary Figure 3: Patient-level ROC curves for OCT-μFR, μQFR, OCT-derived AS%, and QCA-derived DS% to identify FFR ≤0.80**


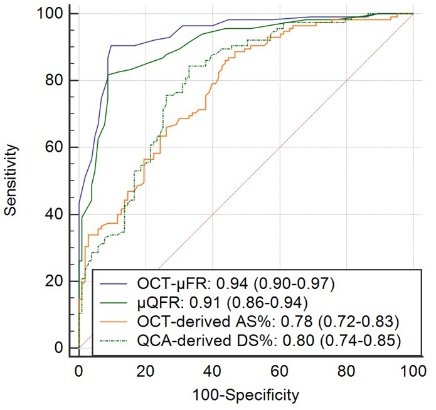


AS%: percent area stenosis; AUC: area under the curves; DS%: percent diameter stenosis; FFR: fractional flow reserve; OCT: optical coherence tomography; QCA: quantitative coronary angiography; μQFR: Murray law-based quantitative flow ratio; OCT-μFR: OCT-modulated Murray law-based quantitative flow ratio; ROC: Receiver operating characteristic.
